# Early Warning Signs for Monitoring Airborne Respiratory Virus Transmission

**DOI:** 10.3390/ijerph22071151

**Published:** 2025-07-20

**Authors:** Qingyang Liu

**Affiliations:** College of Ecology and Environment, Nanjing Forestry University, Nanjing 210037, China; qyliu@njfu.edu.cn

**Keywords:** respiratory viruses, aerosol transmission, early warning, monitoring technology, interdisciplinary research

## Abstract

Airborne respiratory viruses (e.g., influenza, respiratory syncytial virus (RSV), and SARS-CoV-2) continue to pose a serious threat to global public health due to their ability to spread through multiple transmission pathways. Among these, aerosol transmission stands out as a key route, particularly in enclosed environments. However, current monitoring systems have major limitations in sensitivity, standardization, and high time resolution. This study provides a summary of the latest information on the monitoring technologies for respiratory virus aerosols. It discusses the technical and ethical challenges in real-world applications. In addition, this study proposes practical solutions and future development pathways. The aim of this study is to provide theoretical support for building a dynamic, precise, and effective early warning system for monitoring variants of airborne respiratory viruses

## 1. Introduction

Respiratory viruses have always been a significant component of the global disease burden [1,2]. According to data from the World Health Organization (WHO), seasonal influenza causes 3–5 million severe cases worldwide each year, with approximately 290,000–650,000 deaths related to respiratory diseases [3]. The outbreak of the COVID-19 pandemic in 2019 further highlighted the destructive power of airborne viruses [4]. As of 2025, there have been over 750 million confirmed cases and more than 6.8 million deaths globally (https://data.who.int/dashboards/covid19/summary, accessed on 1 June 2025). The difficulty in controlling these viruses lies in their diverse transmission routes, including droplet transmission, aerosol transmission, and contact transmission [5]. Traditional infection control measures, such as wearing masks, hand hygiene, and environmental surface cleaning, mainly target droplet and contact transmission and have limited effectiveness against aerosol transmission [6]

Aerosols refer to respiratory particles with a diameter of less than 100 μm, among which droplet nuclei with a diameter of less than 5 μm can remain suspended in the air for several hours or even longer and can be carried by air currents to distances of tens of meters [5,7]. The global COVID-19 pandemic in 2020 completely changed the scientific community’s understanding of the transmission methods of respiratory viruses [4]. The traditional view held that diseases such as influenza and SARS were mainly transmitted through droplets (>5 μm) and contact [5]. However, the WHO’s updated guidelines in April 2021 explicitly acknowledged that SARS-CoV-2 can be transmitted through aerosols for the first time [5]. This shift in understanding is based on a large number of empirical studies [5,8]. A study pointed out that aerosol particles (1 μm) produced by speaking can remain suspended in still air for up to 9 h, with a transmission distance far exceeding social distancing restrictions [9]. This characteristic makes aerosol transmission the main route of virus spread in closed and poorly ventilated environments, such as hospital wards, public transportation, school classrooms, and office spaces [6,10].

Although aerosol transmission is widely recognized as a significant factor, current monitoring technologies still face several limitations that hinder their broader use in public health surveillance [11,12,13]. One major issue is insufficient sensitivity [14]. In low viral load environments, such as well-ventilated public spaces, many existing detection methods struggle to reliably identify viruses [15,16]. Another challenge is the lack of high spatial resolution [17]. Most monitoring systems require sample collection and lab analysis, delaying results and preventing immediate alerts [18]. Additionally, there is no standardized approach [17,18]. Different research institutions and labs use varying sampling methods, detection techniques, and interpretation criteria, making it difficult to compare data across studies [18]. This study aims to propose practical solutions and future development pathways to address the technical challenges in current monitoring technologies and methodologies of respiratory virus aerosols. To compile this review, a systematic search was conducted using databases such as PubMed, Web of Science, and Google Scholar, focusing on peer-reviewed articles published between 2020 and 2025. Keywords included “airborne respiratory viruses”, “aerosol transmission”, “monitoring technologies”, and “early warning systems”. Studies were selected based on relevance to technical advancements, real-world applicability, and interdisciplinary approaches. Non-English publications and studies lacking empirical data were excluded.

## 2. Sampling Techniques

### 2.1. Active Sampling Techniques

Active sampling uses a pump or similar mechanism to draw air through a collection device, capturing airborne particles and viruses [18]. Some common techniques are impaction samplers and membrane filtration. Impaction samplers use high-velocity airflow to trap particles in a liquid medium (e.g., phosphate-buffered saline) [18,19]. They are highly efficient, especially for larger particles, and the liquid sample can be directly analyzed for viruses. However, their efficiency drops for ultrafine particles (<0.1 μm), and the high-speed airflow may damage viral integrity. These samplers are commonly used in lab research and field monitoring in environments such as in hospitals and laboratories [18]. Membrane filtration captures particles on porous membranes (e.g., polycarbonate or nylon) based on the pore size [18]. It is cost-effective, easy to use, and adaptable to different particle sizes. However, the membrane may physically damage viruses and can clog during operation, reducing the flow rate [20]. This technique is primarily used for qualitative and quantitative analysis of airborne microbes, including viruses (Table 1).

### 2.2. Passive Sampling Techniques

Passive sampling captures airborne particles without external power, relying instead on natural settling and diffusion [20]. The most common passive method is the sedimentation plate technique, where a culture medium-filled plate is exposed to the air, allowing gravity to deposit large particles (>50 μm) [18]. The sedimentation plate method requires no equipment and operates with a low cost [18]. However, its main drawback is its low sensitivity, as it can only collect large particles and cannot reflect the virus content of small particles (especially aerosols) in the air. The sampling time consumes several hours or even days [18]. This method is mainly used for the preliminary screening of microbial contamination in the air, such as in food processing plants and operating rooms, where high hygiene standards are required (Table 1).

### 2.3. Real-Time Monitoring Techniques

Real-time monitoring technologies like aerosol mass spectrometers and electrostatic samplers enable rapid airborne virus detection [21,22]. The aerosol mass spectrometer analyzes particle chemistry through laser ionization [21,22]. This method can detect the chemical compositions of particles in the air in real time and can be used to identify chemical markers related to viruses (such as proteins or nucleic acids). This method cannot directly identify viruses, only inferring their presence through chemical biomarkers [21,22]. While highly effective for research institutions and high-priority venues like biosafety labs and major event spaces, the equipment is costly, operationally complex, and requires specialized maintenance (Table 1).Aerosol mass spectrometers detect virus-specific tracers such as nucleocapsid proteins (e.g., SARS-CoV-2 N-protein) or envelope lipids (e.g., influenza hemagglutinin), which differ from generic aerosol components (e.g., sulfates or nitrates) in their unique mass-to-charge ratios and fragmentation patterns. These biomarkers enable indirect virus identification, though specificity is limited compared with nucleic acid-based methods [21,22].

Electrostatic samplers use charged fields to capture airborne particles on an oppositely charged collection plate, offering high efficiency for small particles while preserving viral integrity [20]. However, these systems require high-voltage operation (posing safety concerns) and post-sampling lab analysis, preventing real-time detection [18,20]. They are particularly valuable for virus transmission research and vaccine development, where particle viability matters more than instant results. Electrostatic samplers preserve viral integrity due to low shear forces, allowing post-sampling analysis via cell culture (e.g., Vero E6 cells for SARS-CoV-2) or viability assays (e.g., plaque reduction neutralization tests). However, high-voltage exposure may reduce infectivity, necessitating validation with surrogate viruses [18,20].

Recent advancements in electrostatic sampler design have addressed safety concerns associated with high-voltage operation [21]. Bhardwaj et al. (2021) [21] developed a low-voltage electrostatic sampler that reduces the operating voltage from 5 kV to 1.5 kV, eliminating the risk of arcing while maintaining an 85% capture efficiency for sub-5 μm particles, comparable to traditional high-voltage systems. This design incorporates a grounded shielding mechanism that diverts excess charge, making it suitable for use in crowded environments such as airports and schools. Field tests in a busy subway station showed that the sampler could operate continuously for 8 h without overheating, capturing viral particles with a recovery rate of 79% (versus 82% for 5 kV systems), confirming its practical viability [21].

Portable real-time monitoring devices have also made significant strides, enabling on-site detection in resource-limited settings. Robotto et al. (2021) [22] validated a handheld electrostatic sampler coupled with a miniaturized RT-qPCR module in a COVID-19 hospital ward. The device, weighing 2.5 kg, completed sampling and detection within 30 min, with 92% concordance with laboratory-based results. Its utility was further demonstrated in a rural clinic in Kenya, where it identified a localized outbreak of respiratory syncytial virus (RSV) 3 days earlier than conventional methods, allowing for timely intervention. These portable systems bridge the gap between laboratory precision and field applicability, making real-time monitoring feasible in diverse contexts.

## 3. Virus Detection

Nucleic acid testing, including qualitative and quantitative PCR, is a primary method for detecting viruses in respiratory diseases. While qualitative PCR is widely used for diagnostics, qPCR offers advantages in quantifying viral load [23,24]. RT-qPCR offers high sensitivity, enabling the detection of trace amounts of viral nucleic acids in samples. Digital droplet polymerase chain reaction (ddPCR) technology provides absolute quantification of viral nucleic acids, allowing for a more precise assessment of viral load in samples [25]. However, these methods are unable to monitor live viruses.

Cell culture remains the gold standard for detecting live viruses [26]. By inoculating collected samples into cell cultures and observing cytopathic effects, researchers can confirm the presence of infectious virus particles. The three-step detection method first intends to screen air samples for viral RNA using qPCR, confirms infectivity through cell culture, and finally verifies virus integrity via electron microscopy [26,27]. This approach successfully differentiated between infectious whole virus particles and non-infectious RNA fragments [26,27]. However, cell culture has limitations; it typically takes 2–7 days, requires specialized lab equipment and trained personnel, and depends on sufficient viral load in samples [26,27]. Low viral loads may go undetected, reducing sensitivity compared with molecular methods like PCR [26,27].

In recent years, some alternative technologies have gradually developed, such as assessing viral activity by detecting the integrity of viral surface proteins (e.g., hemagglutinin protein of influenza virus and spike protein of SARS-CoV-2) [28]. Complete viral proteins usually have immunogenicity and can bind to specific antibodies, while denatured proteins cannot [28]. While using the reverse transcription PCR approach can assess viral activity, it cannot definitively confirm that all detected RNA segments originate from the same intact virus, as fragments may derive from multiple viral particles [29]. For example, primers targeting regions of the viral genome that are prone to damage can be selected. The nucleic acids of complete viruses can be amplified, while the nucleic acids of viral fragments cannot.

CRISPR-based methods demonstrate significant potential for respiratory virus detection, offering advantages in terms of speed, specificity, and field adaptability. By leveraging the CRISPR-Cas system’s programmable nucleic acid targeting, these methods can distinguish even closely related viral strains, such as SARS-CoV-2 Omicron subvariants (e.g., BA.5 and XBB) and influenza A/H3N2 variants, with single-nucleotide resolution [30]. For instance, Cas12a’s collateral cleavage activity enables amplification-free detection, where target binding triggers fluorescent or lateral flow signals, achieving sensitivities as low as 1–10 copies/μL in optimized assays [31]. Recent advancements, such as the RECOGNIZER system, integrate CRISPR with isothermal amplification and microfluidics to simultaneously detect SARS-CoV-2, MERS-CoV, and influenza A/B in 25 min, demonstrating 100% clinical concordance with RT-PCR [30]. Despite these advances, challenges remain. (1) Novel Pathogens: CRISPR relies on predefined guide RNAs, limiting the detection of emerging variants or unknown viruses. (2) Sensitivity: While some platforms (e.g., RT-RPA/CRISPR-Cas12a) achieve near-PCR sensitivity, others (e.g., Cas13a-based lateral flow) require higher viral loads (>300 copies/μL) [30]. (3) Validation: Finally, most studies use contrived samples or small clinical cohorts, necessitating larger real-world trials [30].

In addition, low-concentration environmental detection still faces challenges (Table 2). During air monitoring in hospital wards, the researchers found that prolonged sampling (4 h) improves detection sensitivity (10 copies/m^3^) by accumulating viral load, whereas shorter durations (30 min) require higher ambient concentrations (≥1000 copies/m^3^) for reliable detection [32].To solve this problem, researchers developed a nanoplasmonic sensor that can detect a single virus particle within 10 min through surface-enhanced Raman scattering (SERS) technology, with a sensitivity three orders of magnitude higher than traditional PCR [33]. Emerging technologies have shown great potential in the air monitoring of respiratory disease pathogens. Field-effect transistor (FET) sensors can detect viruses by detecting the changes in electrical signals caused by the specific binding of viruses to the sensor surface, featuring high sensitivity and rapid detection. SERS technology can fingerprint viruses for rapid and accurate detection. Microfluidic chip technology integrates multiple steps such as sampling and detection on a small chip, offering portability, simplicity of operation, and fast detection speed, making it suitable for on-site rapid detection [17]. Commercial systems like Cepheid’s GeneXpert utilize real-time reverse transcription polymerase chain reaction (RT-PCR) technology combined with fluorescence-based detection to achieve rapid and accurate identification of viral RNA. The process begins with the extraction and purification of viral nucleic acids from collected samples. During amplification, target-specific primers and fluorescently labeled probes bind to complementary viral RNA sequences. As the PCR reaction proceeds, the probe is cleaved by the 5′→3′ exonuclease activity of the DNA polymerase, releasing the fluorescent reporter dye from its quencher [24]. This generates a measurable fluorescence signal that increases in direct proportion to the amount of amplified target DNA. The system monitors this fluorescence in real time through optical sensors, allowing for simultaneous amplification and quantification. By comparing the fluorescence signal to internal controls and standardized curves, the system can determine both the presence and viral load of pathogens like SARS-CoV-2 within approximately 45 min [24]. This integrated approach combines the sensitivity of PCR with the specificity of fluorescent probe hybridization, while the closed cartridge design minimizes contamination risks and enables point-of-care testing outside traditional laboratory settings [24].

## 4. Challenges

### 4.1. Technical Challenges

Quality control plays a critical role in air monitoring processes [20]. Preventing contamination during sampling is essential to ensure the accuracy of monitoring results. Maintaining clean sampling equipment is necessary to avoid cross-contamination. At sampling sites, strict protective measures must be implemented to prevent external environmental viruses from contaminating the samples. Currently, the sensitivity of air monitoring technologies in detecting low viral load samples still requires improvement. For example, RT-qPCR typically has a detection limit of 10–100 viral particles per milliliter of air. However, in real-world environments, viral concentrations may fall below this threshold, leading to false negatives. Sample loss during the collection process is another challenge [20]. Due to flaws in sampler design, improper flow rate selection, or filter membrane adsorption effects, some particles may be lost, compromising the accuracy of the test results. For instance, in membrane filtration methods, small particles may pass through the pores without being captured, reducing sampling efficiency [20]. Additionally, certain sampling techniques may fail to collect all viral particles, further impacting the reliability of monitoring data.

Assessing virus recovery rates is critical for accurately quantifying viral load in samples [12]. One effective method involves spiking samples with a known concentration of surrogate viruses (e.g., bacteriophages) during sampling to evaluate losses occurring throughout collection and analysis. The use of internal standards is another key quality control measure. Incorporating these standards helps correct procedural errors and improves detection accuracy [12]. Detecting viable viruses remains the most significant challenge [15]. Traditional cell culture methods take 3–7 days and lack sufficient sensitivity for certain viruses like H1N1 influenza [12]. While the3D organoid aerosol infection model reduces the detection time to 24 h, it still falls short of real-time monitoring needs [34]. Nanopore sequencing can differentiate RNA fragments from intact viruses, but high equipment costs hinder widespread adoption [35]. Cost barriers further limit large-scale monitoring. Many advanced real-time detection systems are prohibitively expensive to purchase and maintain, restricting their use in resource-limited settings. Another major gap is the absence of a unified global data-sharing platform [18]. Without standardized integration of data across regions and institutions, coordinated pandemic surveillance and response efforts remain fragmented.

While emerging detection technologies have shown promise in addressing the shortcomings of traditional methods, their practical application in airborne respiratory virus monitoring is constrained by several critical limitations that require systematic examination.

CRISPR-based detection methods, celebrated for their high specificity in identifying viral nucleic acids, face significant sensitivity barriers. Wang et al. (2025) [30] demonstrated that CRISPR-Cas13a assays for norovirus GII.4 detection exhibit a detection limit of approximately 100 copies/μL, which is an order of magnitude lower than that of RT-qPCR (10–100 copies/mL for most respiratory viruses). This disparity becomes particularly problematic in low viral load environments, such as well-ventilated offices or public transit, where airborne viral concentrations often hover below 50 copies/m^3^. In such scenarios, CRISPR-based methods frequently produce false negatives, undermining their reliability for early warning systems. Furthermore, their dependence on preprogrammed guide RNAs targeting known viral sequences renders them ineffective against novel or uncharacterized pathogens. For instance, during the initial outbreak of COVID-19, CRISPR assays could not be deployed until the SARS-CoV-2 genome was sequenced and specific guide RNAs were designed, resulting in a critical 2–3 week delay in practical application [30]. This limitation highlights a fundamental trade-off between specificity and adaptability, which remains unresolved in current CRISPR-based platforms.

Surface-enhanced Raman scattering (SERS) sensors, lauded for their single-particle detection capability, are plagued by interference in complex real-world environments. Liu et al. [33] reported that in field tests conducted in hospital wards and public transportation hubs, SERS signals from airborne viruses (e.g., SARS-CoV-2) were frequently masked by background particles, including dust, pollen, and microbial debris. Their study showed that in environments with high levels of particulate matter (PM_2.5_ > 50 μg/m^3^), the false positive rate of SERS sensors increased by 42%, as non-viral particles with similar Raman spectra (e.g., proteinaceous aerosols from skin flakes) were misidentified as viral particles. Additionally, the stability of SERS substrates—typically noble metal nanostructures—deteriorates rapidly under humid conditions (relative humidity > 60%), reducing detection sensitivity by up to 30% within 2 h of deployment [33]. These environmental dependencies limit the utility of SERS sensors in uncontrolled settings, where humidity and particulate levels fluctuate widely.

### 4.2. Data Interpretation and Model Construction

In airborne virus monitoring, detecting viral RNA does not confirm the presence of live, infectious viruses [18]. While viral RNA indicates nucleic acid contamination, it cannot distinguish between active viruses and non-infectious genetic material. Therefore, monitoring data must be interpreted with caution. To better assess infection risk, we need to develop concentration-risk correlation models that account for variables such as viral load, exposure duration, and population susceptibility [36]. These models would help predict transmission likelihood. However, these models remain under development and require further research for validation. One critical challenge is establishing actionable warning thresholds by linking airborne viral concentrations to infection risk across different scenarios. Currently, the lack of standardized guidelines and sufficient data makes this difficult. For instance, the WHO has yet to define a safe airborne concentration threshold for SARS-CoV-2. Infection risk is multifactorial, depending not only on the viral concentration but also the exposure time, host immunity, and protective measures (e.g., masking). Effective modeling must incorporate these variables through multivariate analysis to improve accuracy and real-world applicability.

**Figure 1:**
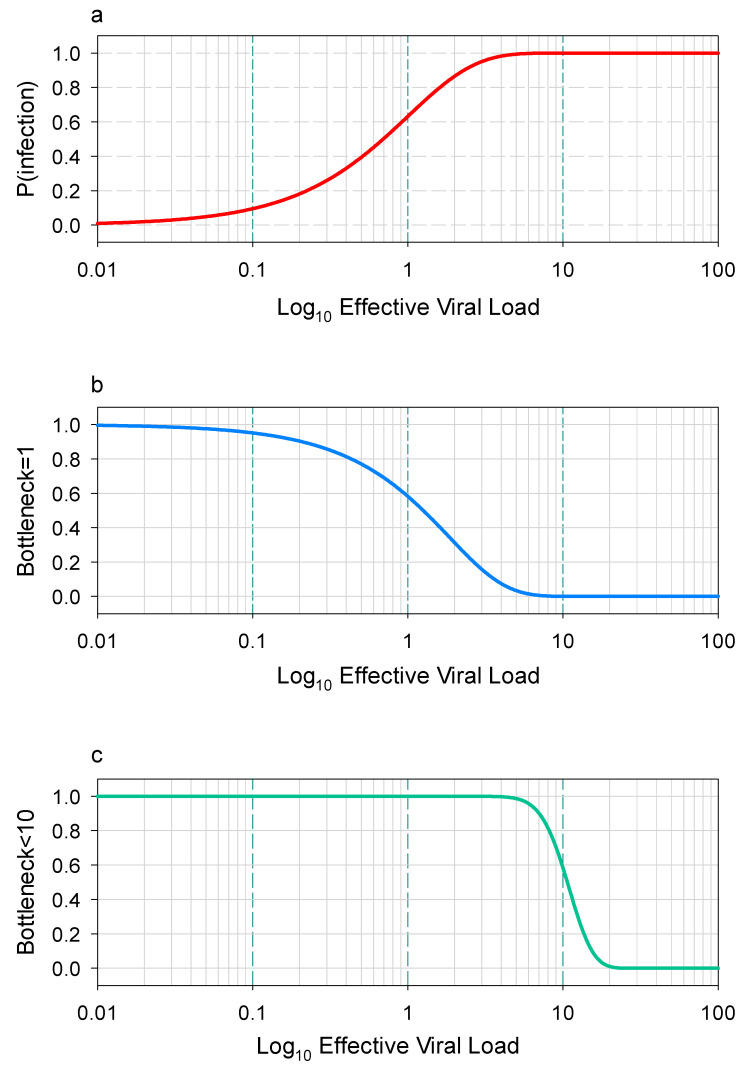
Estimating transmission bottlenecks using a Wells-Riley exposure model. In the Wells-Riley model, the exposure level is represented by the rate parameter of a Poisson distribution, where an exposure value of 1 corresponds to an expected infection by a single virus. (**a**) The probability of infection increases with higher exposure levels. (**b**) Most infections are initiated by just one virus. (**c**) The majority of infections involve 10 or fewer viruses [36]. The data was obtained from an open-access article distributed under the terms of theCreative Commons CC BYlicense.

To bridge the gap between theoretical models and real-world applicability, empirical validation of concentration-risk correlation models is critical. Sinclair et al. (2024) [36] provided evidence for the utility of such models in a study conducted across 12 hospital wards during the 2023–2024 SARS-CoV-2 outbreak using the Wells-Riley model (Figure 1). This model highlights how infection probability increases with viral load, underscoring the need for sensitive monitoring. Their analysis revealed a strong positive correlation between airborne SARS-CoV-2 concentrations and nosocomial infection rates; when viral loads exceeded 10 copies/m^3^, the probability of healthcare worker infection increased by 2.3-fold (95% CI: 1.8–2.9) compared with environments with concentrations below this threshold. Importantly, the study demonstrated that the Wells-Riley model, when adjusted for ward-specific ventilation rates, could predict infection risk with 78% accuracy, far exceeding the 52% accuracy of unadjusted models [36]. This validation highlights the need to incorporate environmental variables into risk assessments [36]. In the Wells-Riley exposure model analysis (Figure 1) [35], this study indicated that infections can be initiated by extremely small amounts of a virus. Even extremely low viral doses in aerosols can lead to successful infection, challenging the notion that high viral loads are always required.

Ventilation in particular emerged as a key modifier of viral transmission dynamics. Firatoglu (2023) [10] showed that natural ventilation in classroom settings reduced airborne viral concentrations by 40–60% compared with mechanically ventilated spaces with recirculated air, leading to a corresponding 35% lower incidence of influenza-like illness. Integrating such empirical data into concentration-risk models allows for more nuanced risk stratification. For example, a viral load of 5 copies/m^3^ in a poorly ventilated office (air change rate < 2 per hour) may pose a higher infection risk than 15 copies/m^3^ in a well-ventilated auditorium (air change rate > 6 per hour). Multivariate analysis, incorporating variables such as exposure duration, population immunity, and mask-wearing prevalence, further refines model accuracy [10].

The establishment of actionable warning thresholds remains contingent on standardized data collection across diverse settings. Currently, discrepancies in sampling methodologies (e.g., flow rates and duration) have led to conflicting reports on “safe” viral concentrations. For instance, studies using 30min sampling with liquid impingers reported SARS-CoV-2 risk thresholds of 50 copies/m^3^, while those using 4h of active sampling with cyclone samplers suggested thresholds as low as 5 copies/m^3^ [18]. Resolving these inconsistencies requires multicenter studies that systematically compare sampling techniques under identical environmental conditions, followed by consensus building among regulatory bodies such as the WHO to define context-specific thresholds [18].

To better assess the relationship between viral quantity and infection risk, the prior studies emphasized the need to develop concentration-risk correlation models [36]. These models integrate variables such as viral load, exposure duration, and population susceptibility to predict transmission likelihood. Such models would help establish actionable thresholds for viral concentrations in air, guiding public health interventions (e.g., ventilation requirements and occupancy limits) based on the minimum infectious dose.

In summary, the amount of virus required to cause infection is surprisingly low (often ≤10 particles), and its impact is modulated by exposure dynamics and host factors [36]. Addressing this issue requires advancing sensitive detection technologies, refining risk models, and distinguishing viable from non-viable viruses to accurately assess and mitigate infection risks.

### 4.3. Ethical and Social Challenges

Equitable access to monitoring technologies is ethically imperative. Currently, 80% of high-throughput airborne virus monitoring systems are concentrated in high-income countries, exacerbating global health disparities [3,4,5]. International initiatives, such as technology transfer programs for low-cost SERS sensors [33] and open-source designs for passive samplers, can help address this gap. For example, a modified version of the sedimentation plate technique, using locally sourced materials, was successfully deployed in rural India to monitor influenza aerosols at 1/20th the cost of commercial systems, demonstrating the potential for low-tech solutions in resource-limited settings.

Moreover, the ethical use of monitoring data requires robust governance frameworks. Regulatory bodies should establish clear guidelines on data retention (e.g., 30 days for non-outbreak periods) and audit mechanisms to prevent unauthorized access [14]. Independent ethics committees, comprising public health experts, legal scholars, and community representatives, can oversee monitoring programs to ensure they align with principles of beneficence, non-maleficence, and justice. By addressing these ethical challenges proactively, airborne virus monitoring can fulfill its potential as a public health tool without compromising individual rights or societal trust.

## 5. Future Research Directions

Fluid dynamics models are able to simulate the diffusion patterns of aerosols in indoor environments and thus can provide a basis for the placement of sampling points and the design of ventilation systems. In addition, the development of novel virus capture materials may improve sampling efficiency and viral viability preservation rates.

The application of machine learning in real-time detection for virus detection may provide a supplement to the current methods (Table 3). Applying machine learning algorithms to analyze and model large volumes of sensor data enhances the sensitivity and specificity of virus detection, such as by training neural network models to identify correlations between chemical signals detected by aerosol mass spectrometers and the presence of viruses, enabling real-time viral alerts.

It is necessary to establish an intelligent early warning system for integrating sensors, data transmission, and an alert platform. When sensors detect virus concentrations exceeding a threshold, the system automatically sends alerts to relevant departments and personnel, allowing prompt implementation of prevention and control measures. Incorporating airborne virus monitoring into national and local public health surveillance systems, establishing unified monitoring protocols and technical standards that define the scope, frequency, and reporting requirements. For instance, conducting regular air monitoring in high-risk settings (such as healthcare facilities, schools, and transportation hubs) to promptly identify signs of viral transmission.

A tiered approach to standardization is proposed to address the current fragmentation in airborne virus monitoring. In the short term (1–2 years), priority should be given to harmonizing core sampling parameters. Based on cross-laboratory comparisons by Kutter et al. (2021) [18], who found that inconsistent flow rates (ranging from 4 to 1000 L/min) accounted for 30–50% of the variability in viral load measurements, we recommend standardizing flow rates to 50–200 L/min for most indoor environments. This range balances efficiency (capturing sufficient particles) and practicality (minimizing solvent evaporation in liquid-based samplers). The sampling duration should also be standardized—1–2 h for high-risk settings (e.g., ICU wards) and 4–6 h for low-risk settings (e.g., shopping malls)—to ensure detectable viral loads while avoiding sample degradation.

In the long term (3–5 years), the establishment of a global data-sharing platform coordinated by the WHO is essential. Integrating virological surveillance with ventilation metrics and standardized sampling methodologies can enhance early warning systems. This platform would integrate data from national surveillance networks, using a standardized format that includes metadata such as the sampling location, ventilation parameters, and detection methodology. Such integration would enable real-time tracking of viral transmission patterns across borders, as demonstrated during the 2022 monkeypox outbreak, where delayed data sharing hindered early containment efforts. To ensure data quality, the platform should mandate adherence to international quality control standards, including the use of surrogate viruses (e.g., bacteriophages) to quantify recovery rates and internal standards to correct for procedural errors [20].

Interdisciplinary collaboration is critical to implementing these standards. Regulatory agencies (e.g., the FDA and EMA) must work with academic institutions to validate standardized methods, while industry partners can develop certified reference materials (e.g., virus-spiked aerosols) to calibrate sampling equipment. Kutter et al. (2021) [18] emphasized that such collaboration reduced inter-laboratory variability by 40% in a pilot study involving 15 European research centers, underscoring the feasibility of this approach. Ultimately, standardized protocols and global data sharing will transform airborne virus monitoring from a fragmented set of local efforts into a unified early warning system capable of mitigating pandemic risks.

## 6. Conclusions

Airborne transmission is a critical pathway for the spread of respiratory viruses, and aerosol monitoring serves as a vital early warning tool with significant implications for epidemic prevention and control. While challenges remain in monitoring technology, data interpretation, and real-world implementation, including limited sensitivity, lack of standardization, and high costs, these issues are expected to be progressively addressed through interdisciplinary research and ongoing technological innovation. Moving forward, airborne virus monitoring technology will advance toward real-time, intelligent, portable, and cost-effective solutions. The integration of cutting-edge technologies like artificial intelligence and sensors will substantially enhance monitoring sensitivity and efficiency. Additionally, the development of a global surveillance network and improved data-sharing mechanisms will strengthen international collaboration and response capabilities in epidemic control. Future systems must differentiate between infectious and non-infectious agents and establish actionable thresholds for viral load. The proposed solution is scientifically viable because it combines viability assays (e.g., rapid 3D organoid cell culture [34] and protein integrity tests [28,33]) with real-time monitoring technologies (e.g., portable electrostatic samplers, microfluidic chips, and CRISPR-enhanced methods) to distinguish infectious from non-infectious viruses [21,30,35]. Empirical studies, such as dose-response models (e.g., 10 copies/m^3^ of SARS-CoV-2 increasing infection risk 2.3-fold [36]) support actionable thresholds, while preventive measures (e.g., HEPA filtration for viable viruses) align with WHO guidelines [4,7]. Interdisciplinary efforts, including standardized protocols [18] and cost-effective tools (e.g., low-voltage samplers [21]) ensure scalability across diverse settings, making this approach both practical and precise for public health responses.

## Figures and Tables

**Table 1:** Active sampling techniques for airborne virus aerosols.

Sampler	Advantages	Disadvantages
Cyclone sampler	Enables size-fractionated aerosol collection and is scalable for parallel sampling, with a flexible duration and high efficiency.	Not intended for infectious virus isolation.
Cascade impactor	This method supports infectious virus isolation and particle size fractionation and offers customizable fraction collection with flexible material selection.	Constrained by anesthesia duration. Labor-intensive and less efficient at low viral concentrations.
Liquid impinger	Designed for infectious virus recovery using multistage liquid impinger technology with integrated aerosol particle size fractionation.	The high liquid volumes risk diluting low-concentration samples below detection limits, while prolonged collection risks solvent evaporation. Additionally, it demonstrates reduced efficiency for sub-0.3 μm particle capture.
Condensation sampler	Designed for low-volume infectious virus collection and recovery.	No size-based aerosol separation and a large physical size.
Filter samplers	With appropriate filter media, this method effectively recovers infectious viruses by maximizing aerosol–surface interactions. The design can be efficiently expanded for high-throughput applications.	This method cannot maintain viral infectivity (filters cause desiccation) and provides no particle size discrimination of aerosols.
Aerosol mass spectrometer	Provides simultaneous quantification of aerosol particle number concentration and chemical components.	This method cannot recover infectious viruses and collects aerosols non-specifically.

**Table 2:** Comparison of major airborne virus monitoring technologies.

Technology	Detection Target	Sensitivity	Time Resolution	Live Virus Discrimination
qPCR	Viral RNA	10 copies/m^3^	2–4 h	No
Viral Culture	Live Virus	100 TCID50/m^3^	3–7 days	Yes
SERS Sensor	Viral Protein	1 particle/m^3^	10 min	Partial
Microfluidic Chip	Intact Virus	10 particles/m^3^	Real-time	Yes

TCID50:50% tissue culture infective dose.

**Table 3:** Challenges and future directions of major airborne virus monitoring technologies.

Challenge	Proposed Solution	Future Direction
Low sensitivity	Nanoplasmonic sensors, microfluidic chips	AI-enhanced signal amplification
Lack of standardization	Unified protocols (WHO and CDC collaboration)	Global data-sharing platforms
High costs	Portable FET/SERS devices	Low-cost CRISPR-based field tests
Viability detection	3D organoid models, protein integrity assays	Rapid viability markers (e.g., viral fusion)

## Data Availability

Not applicable.
